# Developing a guide to climate & health justice education: Process and content

**DOI:** 10.1016/j.joclim.2022.100188

**Published:** 2023

**Authors:** Mita Huq, Sonora English, Heizal Patricia Nagginda, Jon Bonifacio, Thilagawathi Abi Deivanayagam, Rita Issa, Sorcha Ni Chobhthaigh, Priscila de Morais Sato, Hans Mulindwa, Delan Devakumar

**Affiliations:** a Institute for Global Health, University College London, London WC1N 1EH, UK; b Climate Operation Ltd, Kisaasi-Kyanja Road, P.O Box 37705*,* Kampala, Uganda; c Youth Advocates for Climate Action Philippines, College of Science Library, P. Velasquez Street, UP Campus, Diliman, Quezon City, Philippines; d Lancaster Medical School, Faculty of Health and Medicine, Lancaster University, Lancaster LA1 4YG, UK; e School of Public Health, University of São Paulo, Avenida Doutor Arnaldo, 715, São Paulo, Brazil

**Keywords:** Climate justice, Health justice, Climate education, Anti-colonialism, Educational resources, Environmental equity, Lesson design, Curriculum development

## Abstract

Climate justice and health education can address the disproportionate health impacts of climate change on minoritized communities by providing frameworks to build awareness and instigate action on climate-related health inequities. The *Envisioning Environmental Equity Educator's Guide to Climate and Health Justice* provides a framework for educators, activists and health professionals to lead lessons on health and climate justice that center the experiences of those Most Affected People and Areas (MAPA) by climate change. Collaborators in Brazil, the Philippines, and Uganda engaged in stakeholder meetings to assess priorities and needs about climate and health with policymakers, doctors, activists, and students. These meetings informed the product: An educator's guide to climate and health justice that explores their dynamics from an anti-racist, anti-colonial approach. The guide serves as a recommended lesson framework fit with concepts, examples, and activities for educators teaching in primary and secondary learning settings. It is an innovative climate and health justice educational resource that draws on principles of anti-colonialism, critical thinking and consciousness, and engaged pedagogy. It offers a strategy for climate justice communication that targets diverse audiences across climate, health and social contexts by promoting educational approaches that center MAPA experiences, fit for diverse audiences.

## 1 Introduction

Climate justice and health education can address the disproportionate health impacts of climate change on minoritized communities by providing frameworks to build awareness and instigate action on climate-related health inequities [1], [2], [3]. Such educational initiatives are essential as the impacts of climate change materialize along racial, ethnic, and identity lines [4,5]. These inequities are founded by colonial habits [6], making equity central to sustainable climate action decision-making [7]. Nevertheless, this lens appears to be missing in educational spaces that tackle the intersection between climate change and health [8].

While educational materials exist for climate and health, and climate justice modules are abundant, there is a gap in educational resources that convenes health and climate through the lens of anti-coloniality, social justice, and MAPA(most affected peoples and areas) perspectives while taking a more explicit social justice approach [9]. In January 2022, a scoping exercise conducted by the authors found no pre-university educational resources that comprehensively discussed the intersection of climate change, health, and the origins of related injustices in colonialism and oppression. As such, we designed a resource for educators that structures lessons on climate and health justice for late-primary through secondary school settings. This resource distills complex concepts, contextualizes the crisis within colonial and oppressive systems of power, and provides tools and examples that center the perspectives of MAPA, and takes pedagogical approaches relevant to discussing justice and power in diverse learning settings.

## 2 Developing the Guide

The *Envisioning Environmental Equity* (EEE) *Educator's Guide to Climate and Health Justice* is a structured collection of concepts, examples, and activities for educators, activists and health professionals to use when designing lessons on climate and health justice. It equips educators to teach on this nexus while centering MAPA communities using MAPA-relevant and diverse examples, and encouraging reflective, dialogic discussions about the social justice dimension of climate change.

This process was a collective effort by the EEE initiative, a research and public engagement project delivered by University of São Paulo in Brazil (USP), Youth Advocates for Climate Action Philippines (YACAP), Climate Operation (CO) Uganda, and Race & Health, UK. In late 2021, project partners convened under the EEE initiative to secure a funding opportunity to platform MAPA narratives in climate and health justice movements, of which fuels the guide's creation.

### 2.1 Foundational theories & pedagogical approaches

The guide aims to inform climate education with anti-colonial, socially-oriented, justice driven approaches to climate and health education [1]. It draws from pedagogical approaches derived from Paulo Freire [6] and bell hooks [10] to refine content delivery using practices relevant to social justice-oriented learning contexts. To do so, we first developed a humanities-focused climate and health lessons related to injustice, colonialism, and roots of inequality. We then supplemented these lessons with narrative-driven case studies, examples, and activities that interrogate climate-related health inequities in MAPA communities and minoritised communities in global North countries.

We provide pedagogical recommendations for educators using teaching notes provided throughout the guide on how to foster community in the classroom, facilitate dialogic learning environments, and conduct critical reflective activities. These recommendations are informed by Freire's *Pedagogy of the Oppressed,* which emphasizes praxis and dialogue to promote student-led knowledge building that confronts systems of oppression [11]. Concurrently, we draw from bell hooks’ engaged pedagogy that emphasizes the role of community in learning and the teacher's role in establishing learning communities [12]. From this lens, student contributions to the course material, including reactions, connections, and critiques are invaluable. In the context of anti-racism and anti-coloniality, creating a learning experience that is not only critical and creative, but is also generative and empowering was a key principle to the guide's design [13].

### 2.2 Priority setting and content creation

This guide targets educators, activists, and other community educators from Brazil, the Philippines, and Uganda of all ages. Users and educators would have experience leading lessons in institutions and/or with non-governmental (NGO)-led community education settings with learners in late-primary through secondary educational settings.

This target population is diverse in geography, language, educational experience, culture, and institution, meaning their needs will vary across socio- and geopolitical contexts. To identify priority health issues, educational access, relevant content/examples, and perception of climate and health justice, we engaged policymakers, health professionals, activists, and students to discuss their needs, priorities, and perceived gaps in awareness about climate change. Priorities and strategies were further informed by collaborators with prior experience in climate education with communities in their respective countries. These collaborators include climate and health community-based educators, researchers, and health professionals with experience educating and working with local MAPA communities. Subsequent content development priorities can be categorized into three themes: relatability, delivery, and adaptability.

#### 2.2.1 Relatability

Relatability is essential to effective climate change education [14]. Based on input from the stakeholder meetings, and from the teams’ prior experiences, mainstream representations of climate change derive from global North countries and are less relatable to MAPA communities as they lack MAPA-relevant perspectives [15].

To address this, we conceptualized injustice by framing climate change and its related health impacts within colonialism, justice, and its relationship with hierarchical social categorisations (e.g., race, caste, ethnicity) [16]. We also provided instructions, examples, and case studies that encouraged educators to identify locally relevant and relatable examples of discrimination, history, and politics that shaped local climate-related health outcomes. This consideration yielded tangible tools and examples that explore the root causes of climate and health inequities while encouraging each classroom to incorporate local experiences and ideas in the teaching process.

#### 2.2.2 Delivery

Informational gaps exist amongst educators themselves who, though keen to discuss climate justice and health, need more resources about climate and health justice to feel prepared to teach on the subject 17]. Given the target audience diversity, content needed to be tailored to varied levels of education, and easily incorporated into diverse educational cultures.

To address informational gaps, the guide details important concepts relevant to climate and health justice while providing examples from around the world for educators to use and/or adapt to their learners. The format follows a question-and-answer format to inform a logical progression from one concept to the next, and prompts educators to explore discussion questions with students pervasively throughout each section. Educators are encouraged to make use of these questions (or adaptations thereof) to open classroom dialogue per Freire's pedagogical approaches [11] to create space for educators and their students to engage key concepts and invite additional dialogue [18,19].

The guide is a free, printer-friendly resource that will be hosted across collaborator websites. All activities in the guide do not rely on writing materials, presentations, or the internet. The intended mode of delivery thus accounts for varied access to and reliability of internet connection, especially in remote and rural areas most impacted by climate change.

#### 2.2.3 Adaptability

To ensure the guide's usability across diverse contexts, content must be easily translatable and adaptable by educators across diverse learning settings, and include content relevant to key stakeholders. To promote the contents’ transferability across learning contexts, we provided guidance on developing contextually relevant case studies while providing a wide breadth of examples across varied MAPA communities to select from. The guide's adaptability is intended to ensure it can be transferred across contexts while equipping educators with a resource that they can use and reshape according to their needs.

The initial version of the guide is in English as it is the shared language of the collaborators. It was developed to be inclusive of diverse English reading and writing levels, and for ease of translation. However, the guide will be translated into Portuguese, Tagalog, and Luganda for community and institutional education settings in Brazil, the Philippines, and Uganda.

### 2.3 Evaluation and piloting process

**Fig. 1:**
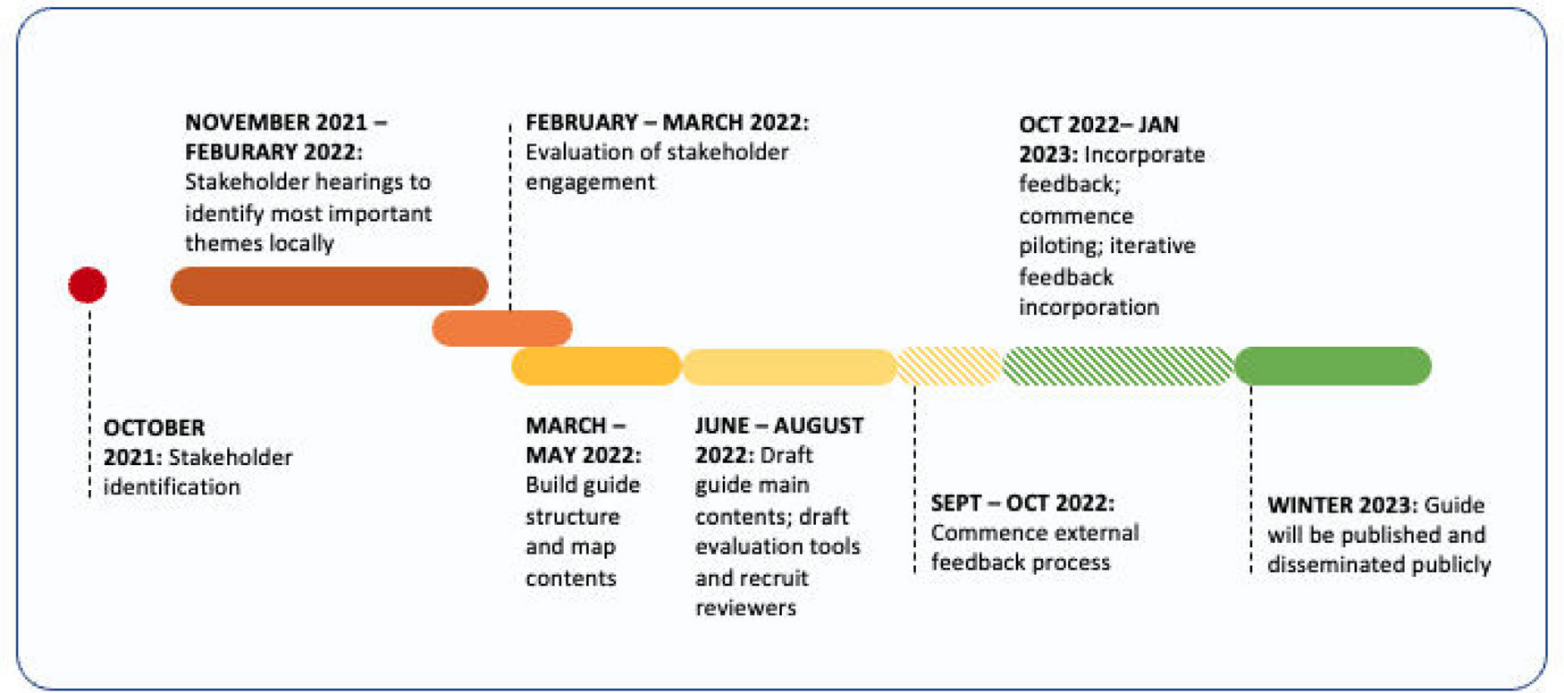
Guide development timeline.

Using a semi-structured qualitative survey that explored predetermined themes around content, accuracy, relatability, and anti-coloniality, the guide was reviewed by an international panel of community educators, institutional educators, climate and health experts, and activists across project sites. Feedback praised the guide for its focus on MAPA perspectives of climate change, and the succinct focus on colonial roots of the crisis. Suggested improvements highlighted a need for further instructions for end-chapter activities, increasing the ratio of examples compared to concepts, and including supporting visuals for educators to use when exploring key concepts. The guide will be piloted at schools in the Philippines and Uganda through existing community relationships affiliated with the respective organization to assess its usability and accessibility (see Fig. 1). Lessons learned from piloting will be integrated into the guide before the first version's publication, with iterations of feedback/adaptations to come.

## 3 Resulting Guide Format

This guide is a detailed course outline that can take between 1–4 months to deliver depending on the educator's expertise and the educational setting (e.g., activist vs. institutional). Its chapters are designed to be presented in succession, however chapters can be extracted and delivered between 2–4 weeks. Each chapter (see Table 1) starts with a content summary and practical recommendations informed by the selected pedagogical approaches. Lesson aims and objectives are presented before the main content presentation, which is delivered in a Q&A format, supplemented with periodic critical thinking questions and summary boxes for reinforcement. Each lesson concludes with an extended case study and proposed discussion activities.

**Table 1:** Guide overview.

**Introduction to the EEE Guide**	Introductory remarks to users Who it is for How to use it
**Chapter 1 - Introduction to climate change, global warming, and humans**	Brief introduction to climate science The relationship between climate change and the humanities
**Chapter 2 - Climate Change and Human Health**	The relationship between climate and health Health inequities across minoritized communities
**Chapter 3 - Climate Change and (In)justice**	Power, oppression, and colonialism in climate-related health injustice Justice principles and their relationship to climate-related health inequity Colonialism as a key contributor to climate change and imbalanced climate decision-making
**Chapter 4 - Moving Towards Justice**	Importance of systems-level changes How to enact system-level changes Critical assessment of proposed climate action for structural change Participating in structural change

## 4 Discussion and conclusion

Reviewers expressed that the EEE Educator's Guide to Climate and Health Justice will be an innovative climate and health justice educational resource that draws on well-established theoretical and methodological approaches. It synthesizes and builds upon existing climate education practices from MAPA settings, sharing the best approaches that have been tried and tested in countries facing some of the worst manifestations of the climate crisis. It also offers a strategy for climate justice communication that targets diverse audiences across climate, health and social contexts by prioritizing modes of delivery, adaptability and relatability.

The guide faces limitations: It lacks audio-visual that would support virtual learning strategies. These were not included in the interest of linguistic diversity due to barriers associated with translating external resources such as videos and podcasts. Coupled with the burden these resources have on areas with limited internet accessibility, such as rural areas heavily impacted by climate change, access and adaptability were prioritized.

Further, the guide attempts to capture a wide target audience. While our iterative feedback process attempts to accommodate this audience, we will also incorporate long-term feedback infrastructure to iteratively collate feedback. Iterations of the guide will be released as feedback is received. Although the original iteration is in English, it will be translated as needed and translations will be made publicly available. This includes an online learning community designed to promote international sharing of translations, examples, methods, and feedback about experiences with both the guide and teaching on climate, health, and justice. This will be hosted on a free project-delivery app and supported by the EEE team.

Our approach tackles both climate and health injustice and the epistemic injustice pervasive in climate communication resources. It encourages educators to create communities in their classrooms that think critically about climate and health injustice and the power structures that fuel them, raising critical consciousness and engagement with rapid and equitable climate action.

Educators can access the guide on the EEE collaborator websites:

*Climate Operation:* https://climate-operation.webflow.io/

*Race & Health:* www.raceandhealth.org

*YACAP:* https://yacap.org/

## Funding

This work was funded by the Wellcome Trust. Grant number: 224687/Z/21/Z. The Wellcome Trust had no influence over the methods followed in this piece, nor the guide.
